# Recurrent Ebolavirus disease in the Democratic Republic of Congo: update and challenges

**DOI:** 10.3934/publichealth.2019.4.502

**Published:** 2019-11-20

**Authors:** Joseph Inungu, Kechi Iheduru-Anderson, Ossam J Odio

**Affiliations:** 1 Master of Public Health Program, College of Health Professions, Central Michigan University, Michigan, United States; 2 Nursing Program, Central Michigan University, Michigan, United States; 3 Department of Internal Medicine, Medical School Hospital, University of Kinshasa, Kinshasa, Congo

**Keywords:** Ebola outbreak, hemorrhagic fever, epidemiology, treatment, prevention

## Abstract

The current Ebolavirus disease (EVD) outbreak in the provinces of North Kivu and Ituri is the tenth outbreak affecting the Democratic Republic of Congo (DRC); the first outbreak occurring in a war context, and the second most deadly Ebolavirus outbreak on record following the 2014 outbreak in West Africa. The DRC government's response consisted of applying a package of interventions including detection and rapid isolation of cases, contact tracing, population mapping, and identification of high-risk areas to inform a coordinated effort. The coordinated effort was to screen, ring vaccinate, and conduct laboratory diagnoses using GeneXpert (Cepheid) polymerase chain reaction. The effort also included ensuring safe and dignified burials and promoting risk communication, community engagement, and social mobilization. Following the adoption of the “Monitored Emergency Use of Unregistered Products Protocol,” a randomized controlled trial of four investigational treatments (mAb114, ZMapp, and REGN-EB3 and Remdesivir) was carried out with all consenting patients with laboratory-confirmed EVD. REGN-EB3 and mAb114 showed promise as treatments for EVD. In addition, one investigational vaccine (rVSV-ZEBOV-GP) was used first, followed by a second prophylactic vaccine (Ad26.ZEBOV/MVA-BN-Filo) to reinforce the prevention. Although the provision of clinical supportive care remains the cornerstone of EVD outbreak management, the DRC response faced daunting challenges including general insecurity, violence and community resistance, appalling poverty, and entrenched distrust of authority. Ebolavirus remains a public health threat. A fully curative treatment is unlikely to be a game-changer given the settings of transmission, zoonotic nature, limits of effectiveness of any therapeutic intervention, and timing of presentation.

## 1. Introduction

The Democratic Republic of Congo (DRC) is facing its tenth Ebolavirus (EVD) outbreak since August 2018. No effective vaccine or curative therapy against this deadly disease is known since its discovery in 1976 in Yambuku, a village in the DRC near the Ebola River. With an estimated 1892 deaths and 2831 confirmed cases on August 11, 2019, the current Ebolavirus disease (EVD) is the second deadly outbreak in history, substantially trailing the 2014–2016 EVD in West Africa in the number of people infected. The ongoing outbreak has surpassed the projected estimates from mathematical modelling studies [1]. The DRC government's response to fight the current outbreak has somewhat contained it within the provinces of Ituri and North Kivu. The detection of one case of EVD in Goma, a city of 2 million people on the border with Rwanda, and the identification of an individual who had traveled while symptomatic from DRC to Uganda and back again before being identified heightened the fear about the risk of EVD transmission to neighboring countries. These two events led the International Health Regulations Emergency Committee on Ebola Viral Disease (IHREC) to declare the current outbreak in the DRC a Public Health Emergency of International Concern (PHEIC) for the world to take notice and redouble its efforts. An outbreak is declared a PHEIC when it poses a public health risk to other countries through the international spread and potentially requires a coordinated international response. The declaration by the PHEIC came during the fourth meeting of the IHREC since the current outbreak was declared last August 2018. The Ebola outbreak in West Africa and the large scale wild Polio virus outbreak were the first ever two events to be declared PHEICs at the same time in 2014 [2].

The increased frequency of EVD outbreaks and the perceived risk for its potential use as a bioterrorism agent underscore the needs for the rapid development of a vaccine [3]. Taking into account the fact that EVD is a threat facing the entire world, and considering the lack of curative drugs against it, the World Health Organization (WHO) developed an ethical framework, the Monitored Emergency Use of Unregistered and Investigational Interventions (MEURI) [4], to establish the criteria that must be met for consenting patients to access investigational therapeutics outside of clinical trials [5]. In the DRC, MEURI helped fast-track unprecedented investigational interventions to test the efficacy of treatment using three antibody-based therapies (mAb114, ZMapp, and REGN-EB3) and one antiviral agent (Remdesivir) among patients with laboratory-confirmed EBOVD [6].

Furthermore, few pharmaceutical companies or research laboratories wanted to seize the opportunity to test promising vaccines, even when it showed efficacy only in non-human primates (NHP). Many EVD vaccine candidates have been described elsewhere [7]–[9]. The Johnson and Johnson experimental Ad26.ZEBOV/MVA-BN-Filo vaccine was one of the few experimental vaccines considered as a second option in addition to the Merck's vaccine shown to have a 97.5% efficacy rate for those who were immunized compared to those who were not [10].

Recent increases in the frequency of natural human Ebolavirus infections and its potential use as a bioterrorism agent makes vaccine development a priority for many nations.

The purpose of this communication is to provide an update about the investigational treatment and vaccines being studied during the current EVD outbreak and discuss the challenges facing the Technical Response Team in the DRC to control the EVD outbreak in the provinces of Ituri and North Kivu in the Congo in 2019.

## 2. Epidemiology

### 2.1. Frequency and Impact of outbreaks

For almost 43 years, EVD and related *Filoviruses* have been repeatedly reemerging across the vast equatorial belt of the African continent causing widespread outbreaks of fatal hemorrhagic fever [11]. EVD case-fatality rate ranges from 25% to as high as 90% in previous outbreaks [12]. The first EVD in 1976 claimed 318 cases and 218 deaths (fatality rate of 88%). Of the 34 EVD outbreaks reported, the highest number (ten) has been in the DRC followed by Uganda with five EVD outbreaks recorded. The 2014 EVD outbreak that began in February 2014 in Guinea was the deadliest Ebola outbreak that spread to Liberia, Sierra Leone, Nigeria, and Senegal [13]. The EVD outbreak reached other continents beyond Africa, with cases reported in Europe (Spain, Italy and England) and North America [14]. Table 1 summarizes the chronology of the EVD outbreak in the DRC.

**Table 1.** Chronology of Ebolavirus outbreak in the Democratic Republic of Congo.

Year of Outbreak	Species	Reported number of cases	Reported number of deaths and percentage of fatal cases	Province
2018 (current)	*Zaire Ebolavirus*	1892	2831	North Kivu
2018	*Zaire Ebolavirus*	54	33 (61%)	
2017	*Zaire Ebolavirus*	8	4 (50%)	Bas UELE
2014	*Zaire Ebolavirus*	65	49 (71%)	Équateur
2012	*Bundibugyo Ebolavirus*	36	13 (36%)	Orientale
2008/2009	*Zaire Ebolavirus*	32	15 (47%)	Kasai Occidental
2007	*Zaire Ebolavirus*	264	187 (71%)	Kasai Occidental
1995	*Zaire Ebolavirus*	315	250 (79%)	Bandundu
1977	*Zaire Ebolavirus*	1	1 (100%)	Orientale
1976	*Zaire Ebolavirus*	317	280 (88%)	Équateur

### 2.2. Types of Ebolavirus

Ebola virus is a member of the *Filoviridae* family of enveloped, negative sense RNA viruses that cause severe hemorrhagic fever in humans and non-human primates (NHPs). In the *Filoviridae* family, three genera have been identified: *Cuevavirus*, *Marburgvirus* and *Ebolavirus*. Five species of *Ebolavirus* species have been identified. They include: (1). *Sudan Ebolavirus* (SEBOV); (2). *Zaire Ebolavirus* (ZEBOV); (3). *Côte d'Ivoire Ebolavirus* (also known and here referred to as *Ivory Coast Ebolavirus* (ICEBOV)); (4). *Reston Ebolavirus* (REBOV) and (5). *Bundibugyo Ebolavirus* (BEBOV)[15]. ZEBOV is the most fatal Ebola virus. Although REBOV and ICEBOV have been found to be pathogenic in NHPs, there has only been one reported non-fatal human case of ICEBOV [16].

### 2.3. Structure of Ebolavirus

**Figure 1.**
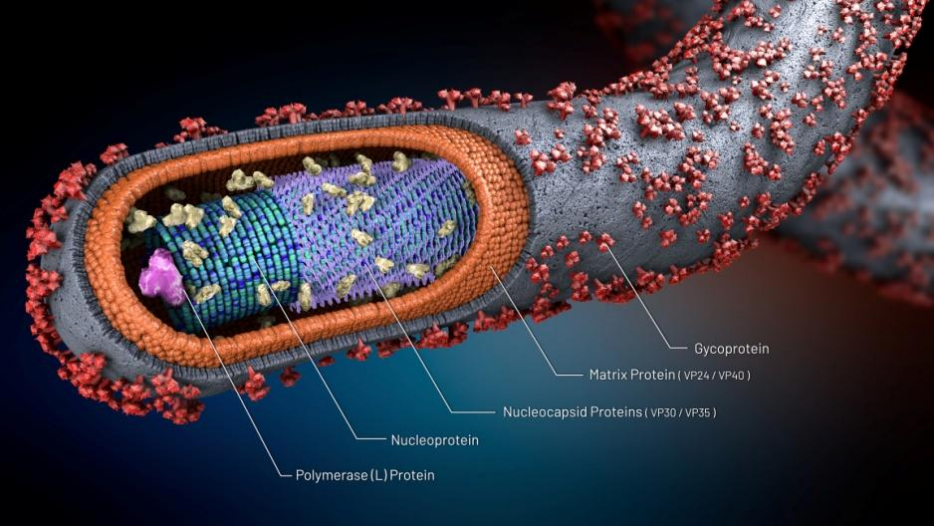
Structure of Ebolavirus.

Ebola virus is a filamentous, enveloped, and negative-sense RNA genome that is approximately 19 kb in length. Each virus genome contains 7 genes that sequentially encode a nucleoprotein (NP), viral proteins (VP35 and VP40), a glycoprotein (GP), two additional viral proteins (VP30 and VP24), and a polymerase (L) (as shown in Figure 1) [3],[11].

### 2.4. Mode of transmission and symptoms

While the precise mechanism of natural virus transmission to humans and non-human primates (NHPs) remains elusive, there are some indications that bats may constitute the natural reservoir and primary source of infection.

Although the precise mechanism of virus transmission to humans and NHPs remains elusive, fruit bats, chimpanzees, gorillas, monkeys, forest antelopes, and porcupines are thought to be possible natural hosts [17]. Ebola virus is introduced into the human population through close contact with the blood, secretions, organs, or other bodily fluids of infected animals. In humans, Ebola virus is transmitted human-to-human via direct contact with bodily fluids (blood, breast milk, saliva, aqueous fluid, urine, and semen) or organs of infected people, or indirectly via contaminated fomites. Health-care workers have frequently been infected while treating patients with suspected or confirmed EVD [18],[19].

EVD begins with vague symptoms (such as fever, fatigue, body aches, vomiting, and diarrhea) that make the infection difficult to distinguish from other infectious diseases such as malaria, typhoid fever, or seasonal flu. Following a short incubation of 2 to 21 days, the condition quickly escalates to involve internal and external bleeding, kidney and liver damage, secondary infections, meningoencephalitis, shock, and hypotension. Death is often due to disseminated intravascular coagulation (DIC) and fibrinolysis, multiorgan, hemorrhage, dehydration and septic shock [20],[21].

### 2.5. Diagnosis

In stable conditions, diagnosis of EVD is performed in appropriately equipped laboratories by staff trained in the relevant technical and safety procedures [22]. Several rapid tests are available to diagnose EVD within only a few days of the onset of symptoms. EVD can be diagnosed by antibody-capture enzyme-linked immunosorbent assay (ELISA), antigen-capture detection tests, serum neutralization test, reverse transcriptase polymerase chain reaction (RT-PCR) assay, and sequencing and genetic analysis [23]. Other diagnostic methods include immunofluorescent (IF) method to detect IgG of Ebola virus [24],virus isolation by culture, and DNA-based fluorescence nanobarcodes methodology [25].

During the 2018 EVD in the Ituri and North Kivu provinces, all laboratories located in Beni, Mangina, Butembo, Komanda, Goma, and Katwa used GeneXpert (Cepheid) PCR to diagnose the disease, whereas laboratories at the National Institute for Biological Research (INRB) in Kinshasa performed the whole-genome sequencing. The real-time sequencing capacity has now been established in Katwa [6].

## 3. Ebola outbreak management

### 3.1. Guiding strategies

Until now, a specific treatment against EVD or a vaccine licensed for use in humans was not available. Effective outbreak control relied on a package of interventions including: (i). Detection and rapid isolation of cases; (ii). Contact tracing; (iii). Extensive population mapping and identification of high-risk areas informed a coordinated effort to screen; (iv). Ring vaccination; (v). Laboratory diagnoses using GeneXpert (Cepheid) polymerase chain reaction as the diagnostic tool; (vi). Safe and dignified burials and (vii). Risk communication, community engagement, and social mobilization [6],[19]. A surveillance-containment strategy using ring vaccination was central to smallpox eradication in the 1970s [26]. Following notification for a laboratory-confirmed case of Ebola (the index case), the study field teams draw a list of contacts using the WHO contact tracing record [27]. An epidemiologically defined ring was formed comprising the index case's contacts and contacts of contacts who may also be at increased risk of EVD [28].

Despite the effort by the Riposte Team, the Ebola outbreak in the Eastern Provinces of the Democratic Republic of Congo (DRC) continued to deteriorate with a large increase in the number of cases The rise in the number of cases was due to security deterioration characterized by the increase in critical security incidents that hampered the Riposte Team's ability to identify and vaccinate contacts successfully. To address security issues and tensions in the community, the Strategic Advisory Group of Experts (SAGE) on Immunization recommended the implementation of two innovative operational strategies to implement ring vaccination [29].

### 3.2. Pop-up vaccination

Rather than setting the vaccination site at the residences of contacts of a given case, vaccination is implemented at an agreed-upon and temporary, protected vaccination site, at a distance from the residence of the contacts. For example, at a health facility or a school.

### 3.3. Targeted geographic vaccination

All the contacts and contacts of contacts of all cases reported in a given village or neighborhood are enumerated and invited for vaccination simultaneously.

A person suspected to be infected with Ebola must be isolated in a single-patient room with the door closed. Healthcare personnel and any person with the potential for exposure to patients and/or to infectious materials, including body substances, contaminated medical supplies and equipment, and contaminated environmental surfaces, should use personal protective equipment (PPE) that covers the clothing, skin, and completely protects mucous membranes [30]. A log should be maintained of everyone who has access to and enters the room. Dedicated medical equipment (preferably disposable whenever possible) should be used for the provision of patient care. Patient's urine, stool, sputum, and blood, along with any non-dedicated, non-disposable medical equipment used for patient care should be cleaned and disinfected (such as laboratory equipment), with a 0.5% sodium hypochlorite solution. Patients who died of EVD were buried promptly with as little contact as possible.

### 3.4. Supportive care and treatment

The provision of clinical supportive care is now the cornerstone for the management of patients suffering from EVD. It consists of hydration, replacement of electrolytes, nutritional support, and maintaining oxygen status and blood pressure. Symptomatic treatment includes the use of antiemetics and antidiarrheal agents to reduce vomiting and diarrhea, and medications to manage fever and pain. Prophylactic antimicrobial agents with intravenous third-generation cephalosporins (e.g., ceftriaxone and cefotaxime) may be administered when secondary bacterial infections and septicemia are suspected [19].

The results of the clinical trial conducted in the Congo will change the management of EVD. For the first time, clinical trial results showed that two Ebola drugs, REGN-EB3, a cocktail of three monoclonal Ebola antibodies made by Regeneron Pharmaceuticals (REGN), and mAb114, a single monoclonal antibody developed by the National Institute of Allergy and Infectious Diseases, improve survival rates.

### 3.5. Vaccination

Several promising vaccine candidates exist [7],[8],[31]. This paper examines only the two vaccines considered during the ongoing outbreak in the DRC.

### 3.6. Vesiculovirus (VSV)-based vaccine candidate

Although not licensed for human use, recombinant vesicular stomatitis virus (rVSV) expressing the *Filovirus* glycoprotein (GP) [ rVSV-ZEBOV-GP ] has been shown to protect macaques from EVD and Marburg virus infections, both prophylactically and postexposure in a homologous challenge setting [32],[33].

VSV is a non-segmented negative-stranded RNA virus that belongs to the family of the Rhabdoviridae. It infects a wide variety of mammalian and insect cells. Infections in humans are asymptomatic or result in a mild febrile illness. The VSV genome is simple and well characterized at the molecular level, which makes the manipulation and production of VSV vaccine vector relatively easy. Replication of the virus occurs within the cytoplasm of the infected cells and is not known to undergo genetic recombination or integration into the cellular genome.

The extremely low percentage of VSV seropositivity in the general population, the lack of serious pathogenicity in humans, and most of all the ability of the VSV to stimulate robust humoral and cellular immune responses against self as well as foreign viral antigens make this virus a potent vaccine carrier [34]. Many researchers studied the use of rVSV as an expression and vaccine vector [35],[36].

Henao-Restrepo, Camacho, Longini, et al. (2016) assessed the efficacy of a single intramuscular dose of rVSV-ZEBOV (2 × 10^7^ plaque-forming units administered in the deltoid muscle) in the prevention of laboratory confirmed EVD. They carried an open-label, cluster-randomized ring vaccination trial in the communities of Conakry and eight surrounding prefectures in the Basse-Guinée region of Guinea, and in Tomkolili and Bombali in Sierra Leone. Of the 4539 contacts and contacts of contacts identified in 51 clusters randomly assigned to immediate vaccination, 2119 were immediately vaccinated. Of the 4557 contacts and contacts of contacts identified in 47 clusters randomly assigned to delayed vaccination 2041 were vaccinated 21 days after randomization. No cases of Ebola virus disease occurred 10 days or more after randomization among randomly assigned contacts and contacts of contacts vaccinated in immediate clusters versus 16 cases (7 clusters affected) among all eligible individuals in delayed clusters. Vaccine efficacy was 100% (95% CI 68.9–100.0, p = 0.0045).

The WHO reported that the rVSV-ZEBOV-GP vaccine candidate (Merck vaccine) showed a 97.5% efficacy rate in the trial in the DRC. Of more than 90,000 people vaccinated, only 71 developed Ebola. Fifty-six of those people presented symptoms fewer than 10 days after being vaccinated, suggesting the vaccine had not yet had time to fully protect them. It takes about 10 days for the immune protection to develop after vaccination.

### 3.7. Adenovirus-Based Vaccine

Another promising vaccine candidate in advanced stages of development was an adenovirus type 26-vectored vaccine encoding Ebola virus glycoprotein (Ad26.ZEBOV), boosted by a modified vaccinia Ankara-vectored vaccine encoding glycoproteins from Ebola, Sudan, and Marburg viruses as well as the nucleoprotein of Tai Forest virus (MVA-BN-Filo) [37]. The adenovirus Ad26.ZEBOV and MVA-BN-Filo is a two-dose vaccine, specifically designed to induce long-lasting protection against all potentially circulating filovirus species [38],[39].

In a phase 1 study of healthy volunteers (n = 87), immunization with Ad26.ZEBOV and MVA-BN-Filo showed seroconversion frequencies of 79%–89% as early as 14 days after prime vaccination with Ad26.ZEBOV. Boosting with MVA-BN-Filo (administered 21 to 57 days later) resulted in sustained elevation of specific immunity. No vaccine-related serious adverse events were reported [37].

To interrupt the chain of transmission of the Ebola outbreak, SAGE recommended adjusting the target population for ring vaccination to include a second and third barrier of immunized individuals around each incident case. Sage recommended the administration of a vaccine other than rVSV-ZEBOV-GP to those at some risk of Ebola in Aires de Santé with cases. WHO reviewed data generated by Ebola vaccine manufacturers on two candidate vaccines: the adenovirus 26 vectored glycoprotein/MVA-BN (Ad26.ZEBOV/MVA-BN) vaccine developed by Johnson & Johnson, and the CanSino-Beijing Institute of Biotechnology (Ad5-EBOV) vaccine.3 SAGE recommends that these lower risk populations would be vaccinated with the J&J vaccine with informed consent [40].

## 4. Challenges that DRC's technical response team faced

### 4.1. The DRC's riposte team faced several challenges

Patient delay to report to the hospital: The first few cases of Ebola were misdiagnosed not only because of the long incubation period of the disease, but also for its ﬂu-like symptoms that mimic other infectious diseases such as malaria, flu, or typhoid fever. Patients wait until the clinical situation deteriorates, usually after failure to respond to anti-malarial and/or antibiotic regimens, before reporting to the hospital [41]. Meanwhile the relatives and close friends of the patients are exposed to the Ebola virus. The delay in the early diagnosis of EVD underscores the importance of having widely available infection control and diagnostic resources in the country.

### 4.2. Weak and decayed health care system

Mismanagement and decades of civil unrest have destroyed the medical infrastructure in the country [42]. The existing hospitals are often ill-equipped to diagnose and care for the EVD. Even the provincial hospitals do not have a biosafety level (BSL)-3 laboratory to safely handle specimens suspected of containing Ebola virus. It took several months before the laboratory capacity was established initially in the Beni, Mangina, and Butembo health zones and subsequently in Komanda, Goma, Katwa, and Bunia. These laboratories are using GeneXpert (Cepheid) polymerase chain reaction to diagnose the disease [6].

### 4.3. The context of war

This is the first Ebola outbreak on record that occurred in a war zone. Dozens of armed groups, the remnants of regional wars sparked by the Rwandan genocide, compete for control of territory and illegal trade of resources such as gold, timber, and illegal drugs. The lack of trust among people makes the uptake of the experimental vaccine problematic. Some people fear that Ebolavirus vaccine could be used to decimate the local population. The lack of trust for health care providers and the population's misinformed views about Ebola undermined the response and contributed to spread the deadly virus [43].

### 4.4. The perception of ambivalence

The perception of ambivalence from the World Health Organization to declare the outbreak a Public Health Emergency of International Concern compounded the issue. Although the 2018–2019 outbreak is the second most deadly on record, it took one full year and four meetings for the WHO experts to declare the outbreak a PHEIC following the detection of a single case of Ebola in Goma. The rising number of deaths in a densely populated area where people survive on trade across the 3 neighboring countries was powerful enough to call the international attention on the outbreak. The early influx of resources and expertise could have affected the transmission of the outbreak.

## 5. Conclusion

Ebola virus disease is a rare but deadly disease in people and non-human primates. The mere fact that its natural reservoir remains elusive makes this condition a serious public health threat. Fortunately, reports from Congolese officials and the U.S. government's National Institute of Allergy and Infectious Diseases (NIAID) about the efficacy of two of the four candidate Ebola drugs—an antibody cocktail called REGN-EB3 developed by Regeneron and a monoclonal antibody called mAb114—brings hope for the future management of this disease.

Although indirect evidences point at some bat species as potential Ebola virus reservoirs, the main bat-maintenance hypothesis has not been confirmed yet. Because the risk of reoccurrence persists, as long as the reservoir of Ebola virus remains unknown, more research is needed to identify the natural reservoir of this deadly virus. African forest ecosystems host a large biodiversity and abound in potential maintenance hosts. How does one puzzle those out? [44].

The Ebola Response Team in the Congo faced daunting challenges. Local personnel have endured death threats and attacks for participating in the Ebola response effort. Increasing the Congolese “ownership” of the outbreak by utilizing Congolese nationals is vitally important. Increased Congolese participation cannot be overemphasized. It will pave the way and ensure the acceptability of the new effective vaccine in this population.

The decayed healthcare infrastructure that survived the colonial time is ill-equipped to diagnose the disease and to deliver supportive care. Point-of-care or other rapid laboratory testing could assist with early diagnosis and early deployment of preventive measures [6].

Because the Merck's vaccine has shown high efficacy rate both pre and post exposure, efforts and money should be invested to support this promising product. Although the investigational Ebola vaccine Ad26.ZEBOV, MVA-BN-Filo (The Janssen investigational Ebola vaccine) has shown outstanding safety and immunogenicity in humans and is highly protective against Ebola challenge in NHPs, demonstration of its efficacy in humans is lacking. The ongoing outbreak offers a great opportunity to complete its phase II in Uganda and start a phase III in the Congo. Using this experimental vaccine candidate in large population under expanded condition is an excellent opportunity to establish its prophylactic protection against Ebola virus disease.
